# Distinguishing transgender DNA methylation

**DOI:** 10.1186/s13148-022-01238-2

**Published:** 2022-02-17

**Authors:** Assam El-Osta

**Affiliations:** 1 Department of Diabetes, Central Clinical School, Monash University, Melbourne, VIC 3004 Australia; 2 Epigenetics in Human Health and Disease Laboratory, Central Clinical School, Monash University, Melbourne, VIC 3004 Australia; 3 Department of Medicine and Therapeutics, The Chinese University of Hong Kong, Sha Tin, Hong Kong SAR; 4 Hong Kong Institute of Diabetes and Obesity, Prince of Wales Hospital, The Chinese University of Hong Kong, 3/F Lui Che Woo Clinical Sciences Building, 30-32 Ngan Shing Street, Sha Tin, Hong Kong SAR; 5 Li Ka Shing Institute of Health Sciences, The Chinese University of Hong Kong, Sha Tin, Hong Kong SAR; 6 Faculty of Health, Department of Technology, Biomedical Laboratory Science, University College Copenhagen, Copenhagen, Denmark

Research into the epigenetic gap of gender incongruence is taking us to unexpected places and a prospective study on transgender people recently published in *Clinical Epigenetics* - “Gender Affirming Hormone Therapy induces specific DNA methylation changes in blood” by Rebecca Shepherd; Ingrid Bretherton; Ken Pang; Toby Mansell; Anna Czajko; Bowon Kim; Amanda Vlahos; Jeffrey D. Zajac; Richard Saffery; Ada Cheung; Boris Novakovic has revealed some surprising results [1].

The major reproductive hormones, estrogen and testosterone, are steroids responsible for driving the development and regulation of the female and male reproductive tissues. When cells are stimulated, more often than not, steroid hormones recognise and bind specific nuclear receptors that can control gene expression. Fundamental to the development and maintenance of sexual phenotype, nuclear hormone receptors are ligand inducible transcription factors that include members of the hormone receptor family. The human estrogen receptor (ER) is one classic example, estrogen activates the translocation of ligand-inducible transcription factors in the nucleus. This transactivation is functionally regulated by nuclear proteins that influence histone modifications on the chromatin template that serve to regulate transcription. Belonging to the same steroid hormone receptor family, the androgen receptor (AR) is a hormone-activated transcription factor that is stimulated by testosterone and its metabolite, 5α-dihydrotestosterone (DHT). Undergoing conformational changes in the cytoplasm, AR dissociates from heat shock proteins to translocate into the nuclear compartment and bind specifically to AR sequence elements in DNA to regulate gene transcription. Like other members of the superfamily, progesterone receptor (PR) when stimulated by progesterone follows a path of ligand-dependent transcriptional activation. The commonality here is the specific recognition of ER, AR and PR transcription factors to bind to response elements in DNA and the involvement of co-regulatory molecules that are capable of regulating histone modifications such as acetylation and the remodelling of chromatin to activate gene transcription. While the shared signalling principles of nuclear hormone receptors are known to precisely coordinate ligand-inducible transcription factors, there is emerging evidence that multiple hormones act in concert to effectively regulate nuclear events and is thought to be central to sexual development, skeletal muscle growth, metabolism and nervous system development. Furthermore, while sex hormones are known to interact with and influence immune response, their capacity to effect changes in DNA methylation remains poorly understood.

In this issue of *Clinical Epigenetics*, the authors have examined the longitudinal impact of gender affirming hormone therapy or GAHT on differential DNA methylation by assessing leukocytes derived from blood of individuals that identified as either, transgender women (*n* = 13) or transgender men (*n* = 13) [1]. The influence of feminising and masculinising hormone therapy at 6 and 12 months were compared to baseline DNA methylation profiles of the same individuals before GAHT. The median age at the commencement of hormone therapy of transgender women was 29 years with an interquartile range (IQR of 22–61) and the median age was 23 years (IQR 21–24) of transgender men.

Masculinising hormone therapy comprised of transdermal or intramuscular testosterone whereas feminising hormone therapy involved estradiol and the inclusion of anti-androgens comprising but not limited to progesterone. Adherence to hormone therapy was assessed by immunoassay measurements of serum testosterone and estrodiol. Comprehensive genome-wide coverage comprised assessment of over 850,000 methylation sites. The EPIC array includes probes that recognise CpG sites located in gene-centric regions such as promoter regions, gene bodies and distal regulatory regions. This assessment of genomic regions revealed GAHT influenced progressive changes in DNA methylation at 6 months that remained or advanced at 12 months for transgender women and transgender men when compared to baseline methylation. Close examination of the longitudinal impact emphasises divergent methylation directions with time. Exclusive hypomethylation clusters were associated with feminising GAHT whereas unambiguous hypermethylation clusters were associated with masculinising GAHT. While this opposing methylation pattern expanded after 12 months of GAHT, transient methylation clusters were also identified, showing consequential gains of DNA methylation at 6 months and returns to baseline methylation levels at 12 months. The influence of GAHT on DNA methylation is clearly dynamic and unmistakably complicated.

Interestingly, the prospective analyses highlighted previously unrecognised DNA methylation signatures. For example, GAHT dramatically influenced immune cell DNA methylation in an age-dependent and sex-specific manner. Relative to baseline indices, feminising GAHT reduced DNA methylation at the 3’ UTR of the *VMP1* gene at 6 and 12 months. Methylation robustness was also observed with masculinising GAHT identifying reduced methylation of the *PRR4* promoter was indeed sex-specific and also considered to be age-dependent. In fact, close examination of array probes in and around this region confirmed reduced methylation in people assigned male at birth when compared to people assigned female at birth.

Studies in gender dysphoria and the science of GAHT haven’t always been inclusive. The size of the transgender population remains for the best part—imprecise and uncertain, related in part by the accuracy of census data. Transgender people also grapple with barriers to healthcare and the dilemma of engagement with clinical care services. That inequality in fundamental research presents complicated challenges understanding health outcomes in adults receiving GAHT. While the current longitudinal cohort involved a small number of transgender individuals, the study did not involve longitudinal age-matched cisgender representation [2]. Nonetheless, forging ahead without a larger prospective cohort or GAHT-free cisgender representation would have meant abandoning informative methylation indices which the article systematically describes for transgender women and transgender men. Due consideration to statistical and power estimates, the article published in *Clinical Epigenetics* remains informative.

The limitation of sample size aside, the authors of the article identify a vast number of differentially methylated sites with each one carrying valuable information. The challenge now is to understand their biological function in the context of autoimmune legacy and future infection risk. The most direct mechanism by which DNA methylation could influence gene expression is altering the binding sites of transcription factors. Nuclear receptor inducible transcription relies on response elements that are also subject to DNA methylation and could provide a potential mechanism for stable transcriptional control. An attractive alternative mechanism of transcriptional control could also be independent of nuclear response elements. Pioneering examples exist in the regulation of transcription such as MeCP2, a reader protein that specifically binds methylated CG dinucleotides. While the functional importance of DNA methylation on the nuclear receptor-signalling axis in the context of GAHT remains unclear, it may serve as a code to integrate complex pathways regulating immune response. Chromatin is not only a central integrator of nuclear hormone receptor action; chromatin is also directly influenced by DNA methylation and nowhere is this complexity more evident than by the action of reader proteins such as MeCP2 on gene function. Indeed, it is likely that how GAHT influences DNA methylation mediated events, could unambiguously merge other prime candidates. For example, DNA methylation is often reciprocally regulated by the action of enzymes responsible for writing and erasing post translational modifications on histone and non-histone proteins. Indeed, the assembly of specialised chromatin structures on methylated DNA could help explain the capacity of GAHT to effectively regulate gene behaviour. Future research to systematically pinpoint the regulatory determinants may offer opportunities to understand complex immune response pathways. With an optimistic outlook on future research the identification of methylation-dependent gene expression represents a first essential step to dissect the role of GAHT in signaling networks.

Population studies are challenging, biology is complicated and the science of GAHT epigenetics is rarely simple, but studies like this offers hope and deserve attention that fundamental research can shift and readily pivot to translate transgender health and individual care.
